# Single and Combined Effects of Aged Polyethylene Microplastics and Cadmium on Nitrogen Species in Stormwater Filtration Systems: Perspectives from Treatment Efficiency, Key Microbial Communities, and Nitrogen Cycling Functional Genes

**DOI:** 10.3390/molecules30071464

**Published:** 2025-03-26

**Authors:** Cong Men, Zixin Pan, Jiayao Liu, Sun Miao, Xin Yuan, Yanyan Zhang, Nina Yang, Shikun Cheng, Zifu Li, Jiane Zuo

**Affiliations:** 1 Beijing Key Laboratory of Resource-Oriented Treatment of Industrial Pollutants, School of Energy and Environmental Engineering, University of Science and Technology Beijing, Beijing 100083, China; mencong@ustb.edu.cn (C.M.);; 2 State Key Laboratory of Regional Environment and Sustainability, School of Environment, Tsinghua University, Beijing 100084, China; 3 State Key Laboratory of Iron and Steel Industry Environmental Protection, Central Research Institute of Building and Construction, Co., Ltd., MCC Group, Beijing 100088, China; 4 Institute of Environment and Ecology, Tsinghua Shenzhen International Graduate School, Shenzhen 518055, China

**Keywords:** microplastics, stormwater filtration system, nitrogen removal, functional genes, microbial community

## Abstract

Microplastics and heavy metal contamination frequently co-occur in stormwater filtration systems, where their interactions may potentially compromise nitrogen removal. Current research on microplastics and Cd contamination predominantly focuses on soils and constructed wetlands, with limited attention given to stormwater filtration systems. In this study, the single and synergistic effects of aged polyethylene microplastics (PE) and cadmium (Cd) contamination in stormwater infiltration systems were investigated from perspectives of nitrogen removal, microbial community structures, and predicted functional genes in nitrogen cycling. Results showed that PE single contamination demonstrated stronger inhibition on NO_3_^−^–N removal than Cd. Low-level PE contamination (PE content: 0.1% *w*/*w*) in Cd-contaminated systems showed stronger inhibitory effect than high-level PE contamination (PE content: 5% *w*/*w*). The mean NO_3_^−^–N removal efficiency under combined Cd50 (Cd concentration: 50 μg/L) and PE5 contamination during the sixth rainstorm event was 1.04 to 34.68 times that under other contamination scenarios. Metagenomic analysis identified keystone genera (*Saccharimonadales*, *Enterobacter*, *Aeromonas*, etc.), and critical nitrogen transformation pathways (nitrate reduction to ammonium, denitrification, nitrogen fixation, and nitrification) govern system performance. PE and Cd contamination effects were most pronounced on nitrification/denitrification enzymes beyond nitrite oxidase and nitrate reductase. These mechanistic findings advance our understanding of co-contaminant interactions in stormwater filtration systems.

## 1. Introduction

Stormwater filtration systems, as the core component of sponge city facilities, play an irreplaceable role in mitigating urban flooding, recharging groundwater resources, and controlling nonpoint source pollution [1,2]. With the growing emphasis on water resource recycling, it has become critical to remove nitrogen pollutants in stormwater runoff based on these filtration systems [3]. Dissolved nitrogen species (especially ammonium nitrogen and nitrate nitrogen) in stormwater runoff can trigger eutrophication in receiving waters or lead to groundwater nitrate contamination when inadequately treated, posing significant risks to both ecosystem integrity and public health [4,5]. Consequently, elucidating the nitrogen removal efficiency and underlying nitrogen transformation mechanisms within stormwater filtration systems is essential for advancing sustainable urban water management strategies.

Microplastics, as emerging pollutants, are ubiquitously present in environmental systems, including stormwater runoff and filtration systems [6,7]. These particles interfere with the nitrogen cycle through altering substrate pore architecture, modifying adsorption–desorption dynamics, and impairing microbial metabolic functions [8,9]. Microplastics significantly modify media porosity and hydraulic conductivity, which governs dissolved oxygen diffusion patterns and substrate transport processes, thereby altering the vertical spatial differentiation between nitrification and denitrification zones [10,11,12,13]. Chemical additives leached from microplastics compete for adsorption sites with nitrogen species, and induce structural reorganization of microbial communities, ultimately regulating the abundance of nitrogen-cycling functional genes and associated enzymatic activities [14,15]. However, current research primarily focuses on soils and constructed wetlands, and the influencing mechanism of microplastics on nitrogen transformation processes in stormwater filtration systems remains critically understudied [16,17,18].

Cadmium (Cd) is a typical toxic pollutant in stormwater runoff. Various human activities, such as industrial activities and traffic pollution, can contribute to the accumulation of Cd in stormwater runoff, and its event mean concentration could be up to 900 μg/L [19,20,21]. The accumulation of Cd in stormwater runoff may influence nitrogen migration and transformation in filtration systems through competition for adsorption sites, destruction of cell membrane integrity, and interference with electron transport chains [22,23,24]. Significantly, microplastics and Cd are commonly coexisted in stormwater runoff and filtration systems, where they exhibit distinct interactions [25,26,27]. The hydrophobic surfaces and high specific surface area of microplastics facilitate Cd adsorption and enrichment, functioning as contaminant vectors that modify Cd bioavailability [28,29]. Concurrently, Cd may accelerate microplastic aging processes and additive release, inducing synergistic ecotoxicological effects [30,31]. These interactions potentially reshape microbial community structures and nitrogen metabolic networks within filtration systems, ultimately affecting nitrogen removal efficiency [32]. Existing studies on the combined effects of microplastics and Cd on nitrogen removal have primarily focused on soil contamination [32,33,34]. To our knowledge, the mechanistic understanding of how combined contamination of microplastic and Cd affects nitrogen removal performance and regulates functional microbial genes associated with nitrogen-cycling processes in stormwater filtration systems remains unexplored.

In this study, aged polyethylene microplastics (PE) and Cd were selected as target contaminants to investigate their combined effects on nitrogen transformation in urban stormwater filtration systems. Polyethylene is one of the most prevalent conventional plastics in urban environments, and polyethylene microplastics are widely detected in stormwater runoff and undergo aging processes in actual environments. The main objectives of this study were to analyze the single and combined effect of aged polyethylene microplastics and cadmium in stormwater filtration systems in perspectives from nitrogen removal, key microbial communities, and predicted functional genes in nitrogen cycling. It was hypothesized that the combined effect of PE and Cd in filtration systems was different from the single effect of PE or Cd. This work provides novel insights into the effects of microplastics and their combined effects with heavy metal stress on biogeochemical processes, addressing a critical knowledge gap in urban stormwater management strategies under emerging contaminant coexistence.

## 2. Results and Discussion

### 2.1. The Cd Takeaway Efficiency Under Aged PE Contamination in Filtration Systems

**Figure 1:**
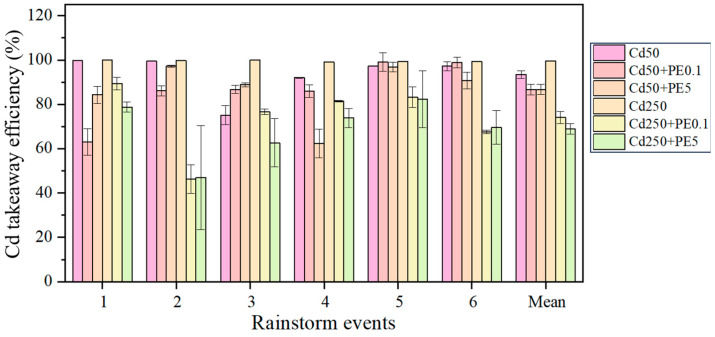
The takeaway efficiency of Cd in filtration systems.

PE showed an adverse effect on the physical takeaway of Cd in filtration systems (Figure 1). For stormwater runoff contaminated by 250 μg/L Cd, the mean takeaway efficiency of Cd in filtration systems without PE was 99.59%, whereas those in filtration systems containing PE were no higher than 74.05%. The adverse effect on Cd takeaway was strengthened with increasing PE content. The mean Cd takeaway efficiency in filtration systems containing 0.1% PE and 5% PE was 80.34% and 77.85%, respectively. PE can change the physical and chemical properties of the filtration system (e.g., porosity, water content, and organic/inorganic matters), which directly influence Cd transport dynamics. Zhang et al. [35] also found that the addition of PE into soil enhanced the mobility of Cd via mitigating the soil-adsorbing capacity, and the PE-induced decrease in Cd adsorption and increase in Cd desorption were more pronounced at higher PE doses. This aligns with our observation of diminished takeaway efficiency under PE contamination. Additionally, inorganic elements detected in PE (e.g., Ca, Mg, Na, As, Zn; Table S1) may compete with Cd for adsorption sites in the filtration media [36], further limiting Cd retention and promoting its physical takeaway. PE-induced changes in media characteristics, such as porosity and aggregate stability [37,38], likely altered hydraulic pathways and enhanced Cd leaching during simulated rainstorms. These combined effects highlight the dominance of physical takeaway mechanisms (e.g., desorption and hydraulic flushing) over chemical stabilization in governing Cd fate within PE-contaminated systems.

### 2.2. The Single and Combined Impact of PE and Cd on Nitrogen Removal

**Figure 2:**
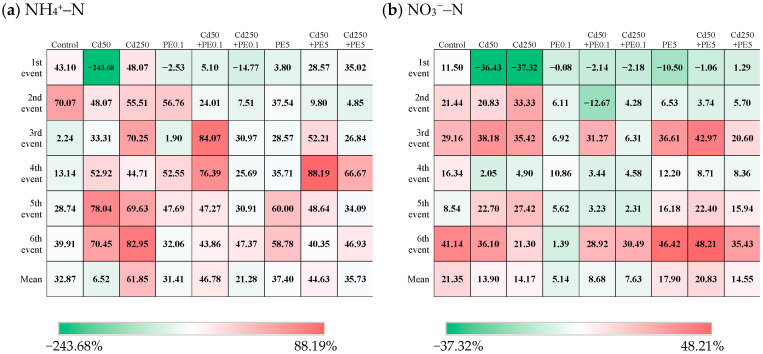
The removal efficiency of nitrogen species in filtration systems (Control represented control groups; Cd50 and Cd250 represented the concentration of Cd being 50 μg/L and 250 μg/L, respectively; PE0.1 and PE5 represented the content of PE being 0.1% and 5%, respectively; + represented the combined contamination of Cd and PE).

Compared with the control group, the removal efficiency of NH_4_^+^–N in the third to sixth rainstorm events were enhanced under both the single and combined contamination of PE and Cd (Figure 2a). The mean removal rate of NH_4_^+^–N from the third to sixth rainstorm events was 21.01% without PE and Cd addition, whereas it was 33.74% to 66.89% for other addition scenarios. For the single-contamination scenarios, PE0.1 and PE5 were less beneficial than Cd50 and Cd250 for the removal of NH_4_^+^–N. The removal efficiency of NH_4_^+^–N in the sixth rainstorm events under Cd contamination was 1.20 to 2.59 times that under PE contamination. The removal efficiency of NH_4_^+^–N under single contamination of Cd250 was higher than that of Cd50. Cd might react with anions (e.g., PO_4_^3−^) in the filtration media to form surface precipitates, which can increase the negative surface charge density of the media, thereby enhancing the adsorption of NH_4_^+^–N in the system [39]. The impact of Cd was influenced by PE. The removal efficiency of NH_4_^+^–N under the combined contamination of Cd250 and PE was lower than that of Cd50 and PE, which contrasts with the trend observed under single-Cd-contamination scenarios. The mean removal efficiency of NH_4_^+^–N under the combined contamination of Cd50 and PE0.1 was 46.78%, whereas that of Cd250 and PE0.1 was only 21.28%.

Overall, both the single and combined contamination of PE and Cd posed adverse effects on the removal of NO_3_^−^–N (Figure 2b). For the single-contamination scenarios, the mean removal rate of efficiency of NO_3_^−^–N under PE0.1, PE5, Cd50, and Cd250 contamination was 5.14%, 17.90%, 13.90%, and 14.17%, respectively. This suggested that the adverse effect intensities of PE and Cd changed with their contamination levels, and PE might lead to stronger adverse effect than Cd. Among all scenarios, mean removal efficiency of NO_3_^−^–N under the single contamination of PE0.1 was the lowest, followed by that under the combined contamination of PE0.1 and Cd (both Cd50 and Cd250). The contamination level of PE was more influential than that of Cd for the removal efficiency of NO_3_^−^–N. By comparing the contamination of PE0.1 and PE5, no matter single contamination or its combined contamination with Cd, the removal efficiency of NO_3_^−^–N was increased with the intensified contamination of PE (from PE0.1 to PE5). Furthermore, the removal efficiencies of NO_3_^−^–N under the single contamination of Cd were higher than those under their combined contamination with PE0.1, whereas lower than those under their combined contamination with PE5. This indicated that high contamination of PE might lead to a positive effect on the removal of NO_3_^−^–N for Cd-contaminated filtration systems, whereas light contamination of PE might lead to adverse effects. The mean removal efficiency of NO_3_^−^–N under the combined contamination of Cd50 and PE5 was the highest among all contamination scenarios. The mean removal efficiency of NO_3_^−^–N under the combined contamination of Cd50 and PE5 in the sixth rainstorm events was 48.24%, which was 1.04 to 34.68 times that under other contamination scenarios. The negative removal rate of efficiency of NO_3_^−^–N in the first rainstorm event might be related to the NO_3_^−^–N release from filtration media [40].

### 2.3. Microbial Communities Under Single and Combined Contamination of PE and Cd

The sequencing of samples analyzed was reliable with the goods coverage index being over 99% for each sample. Chao 1, Shannon, and Simpson were calculated to reflect the richness and diversity of microbial communities, and higher values of these indices generally indicated higher richness and diversity (Table 1). On the 10th day, both single and combined contamination of PE and Cd decreased the richness and diversity. The value of Chao 1 of the control group on the 10th day was 754.06, which was 1.46 to 2.13 times that of other contamination scenarios. On the 30th day, both the single contamination of PE0.1 and its combined contamination with Cd50 increased the richness and diversity, with the value of Chao 1 being 1.28 and 1.14 times that of the control group. The value of Chao 1, Shannon, and Simpson under PE0.1 contamination were all higher than those under PE5 contamination. This indicated that low contamination of PE might be beneficial to the richness and diversity of the microbial communities, whereas high contamination of PE might be harmful. Among all contamination scenarios, the richness and diversity under the single contamination of PE0.1 was the highest during the whole experiment period.

**Figure 3:**
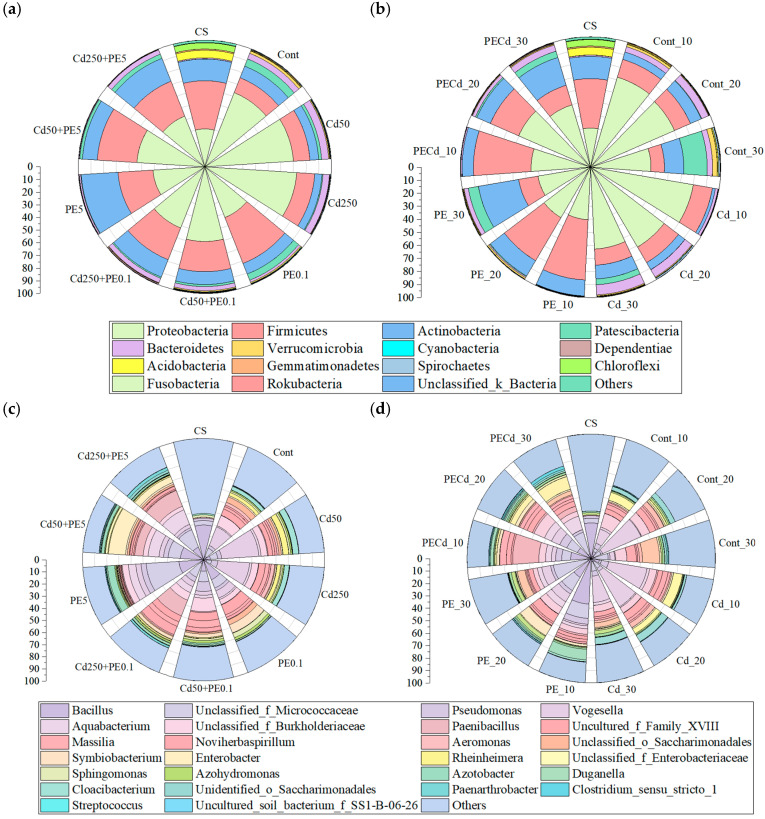
Relative abundance of microbial communities: (**a**) phylum level under different contamination scenarios; (**b**) phylum level in different reaction time; (**c**) genus level under different contamination scenarios; (**d**) genus level in different reaction time (CS represented relative abundance before the first rainstorm event; Cont represented control groups; Cd50 and Cd250 represented the concentration of Cd being 50 μg/L and 250 μg/L, respectively; PE0.1 and PE5 represented the content of PE being 0.1% and 5%, respectively; + represented the combined contamination of Cd and PE; _10, _20 and _30 represented the 10th, 20th, and 30th day).

There were 38 phyla and 880 genera in all samples collected in filtration systems, and the variation in top 15 dominant phyla and top 29 dominant genera (relative abundance > 1%) were analyzed (Figure 3). Phylum Proteobacteria, Firmicutes, and Actinobacteria accounted for 91.28% of all microbial communities at average. Mean relative abundance of Genus *Bacillus*, *Unclassified_f_Micrococcaceae*, and *Vogesella* ranged from 9.81% to 12.12%, and that of Genus *Pseudomonas*, *Aquabacterium*, *Unclassified_f_Burkholderiaceae*, *Paenibacillus*, *Uncultured_f_Family_XVIII*, *Massilia*, *Noviherbaspirillum*, and *Enterobacter* were also higher than 2%.

PE and Cd showed a different effect on the microbial community. Compared with other phyla, Proteobacteria and Bacteroidetes showed a stronger tolerance to Cd but showed a weaker tolerance to PE. Genus *Vogesella*, *Aeromonas*, *Rheinheimera*, *Cloacibacterium*, and *Unclassified_f_Rhodocyclaceae* belong to Proteobacteria, and Bacteroidetes also showed this kind of trend. The relative abundances of Proteobacteria under Cd single contamination (>70.14%) were higher than that in the control group (62.97%), whereas their relative abundances under PE single contamination (<43.12%) were lower than that in the control group. Yu et al. [41] also found that higher concentrations of Cd significantly increased the abundance of Proteobacteria. Liu et al. [42] found that Proteobacteria and Bacteroidetes were Cd-tolerant in alkaline soils. Other studies also showed that *Aeromonas* had better adaptation to Cd tolerance test than other genera, such as *Pseudomonas* and *Enterobacter* [43,44]. Opposite to Proteobacteria and Bacteroidetes, Phylum Firmicutes and Actinobacteria showed stronger tolerance to PE, whereas showed weaker tolerance to Cd. *Bacillus* and *Unclassified_f_Micrococcaceae* were two genera belonging to Firmicutes and Actinobacteria, respectively, and their relative abundance also showed the same trend as their phylum. The mean relative abundance of Firmicutes under PE single contamination was higher than 27.62%, whereas that under Cd single contamination was lower than 14.30%. The mean relative abundance of *Bacillus* under PE single contamination (>17.57%) was higher than that under Cd single contamination (<5.70%). Qiu et al. [45] also found that PE enhanced the abundance of Actinobacteria and reduced that of Proteobacteria in soils. Some studies also found that *Bacillus* and Micrococcaceae were potential engineering bacteria, which can contribute to the biodegradation of PE [46,47,48].

The combined effect of PE and Cd varied for different microbes and changed with the contamination level of both PE and Cd. For *Bacillus* and *Lysobacter*, the relative abundance under combined effect of PE and Cd were higher than PE single contamination and Cd single contamination, and the relative abundance decreased with the intensified Cd contamination. On the contrary, the relative abundance of *Aquabacterium* and *Paenibacillus* under the combined effect of PE and Cd decreased with the intensified Cd contamination. For *Unclassified_f_Micrococcaceae*, its relative abundance under the combined effect of PE0.1 and Cd (8.84% and 12.01%) was higher than that under the PE0.1 single contamination (7.53%), whereas its relative abundance under the combined effect of PE5 and Cd (10.23% and 15.39%) was lower than that under the PE5 single contamination (27.16%). The combined effect of PE and Cd on the microbial community might be influenced by the interaction between PE and Cd. PEs, characterized by their large specific surface area and hydrophobicity, have the capacity to adsorb heavy metals such as Cd, thereby influencing the migration of Cd [49]. The adsorption of Cd by microplastics can alter the bioavailability of the heavy metal, subsequently impacting the microbial community structure [50,51].

The effects of PE and Cd contamination on the temporal change in microbial community were different among different phyla and genera. The abundance of Proteobacteria and its genus *Unclassified_f_Micrococcaceae* both increased with the reaction time under all contamination scenarios. The mean relative abundance of Proteobacteria on the 10th day was 8.71% under the combined contamination of PE and Cd, whereas its value on the 30th day was increased to 22.15%. The mean relative abundance of Actinobacteria under Cd single contamination decreased with time. However, the abundance of Firmicutes and its genus *Bacillus* both decreased with the reaction time under the combined contamination of PE and Cd. The mean relative abundance of Firmicutes on the 10th day (44.23%) was 2.81 times that on the 30th day (15.75%) under the combined contamination of PE and Cd. The microbial community succession might be partly influenced by the stability and bioavailability of Cd and other metals released from PE [52,53]. The colonization of microbes on PE might exert a strong influence on the microbial community, and different microbial groups might employ varying ecological strategies [54]. The hydrodynamic process of the alternation of wetting and drying in the filtration system might be another factor influencing the microbial community succession [55].

**Figure 4:**
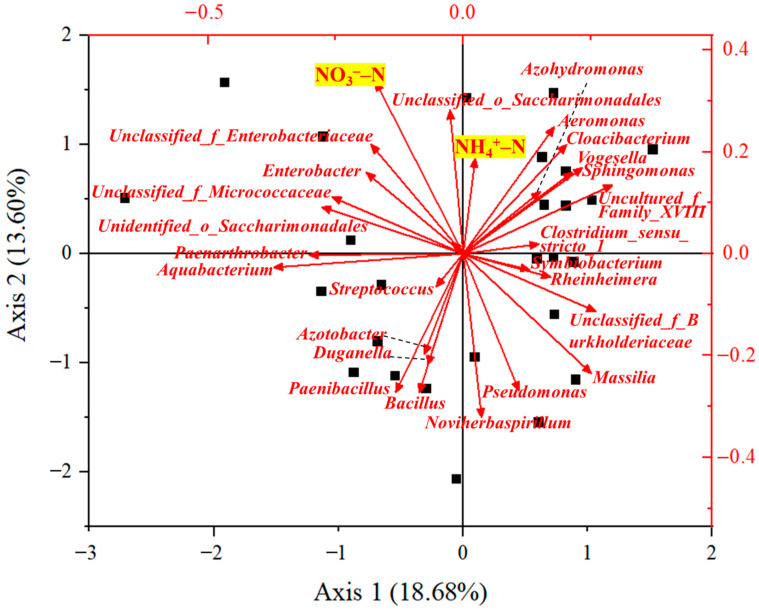
Relation between removal efficiencies and abundance of microbial communities. NH_4_^+^–N and NO_3_^−^–N were highlighted.

Genera beneficial to the removal of NH_4_^+^–N and NO_3_^−^–N were identified (Figure 4). *Unclassified_o_Saccharimonadales* was beneficial to the removal of both NH_4_^+^–N and NO_3_^−^–N, and *Unidenrified_o_Saccharimonadales* was also beneficial to the removal of NO_3_^−^–N. *Saccharimonadales* is the main denitrifying bacteria [56], and *Saccharimonadales* was found to be the most abundant bacterium in the partial denitrification/anerobic ammonia oxidation (anammox) (PD/A) in a moving-bed biofilm reactor [57]. *Unclassified_f_Enterobacteriaceae* and *Enterobacter* were found to be positively correlated with the removal of NO_3_^−^–N. This might be because *Enterobacteriaceae* can produce key enzymes of the nitrogen assimilation pathway [58]. For the removal of NH_4_^+^–N, *Aeromonas* and *Cloacibacterium* were contributable. *Aeromonas* has been isolated from activated sludge and river sediment in some studies, and it was found that *Aeromonas* had high denitrification performance under low-temperature conditions [59]. *Cloacibacterium* was found to be the additionally distinct bacteria which primarily drives the removal of total nitrogen and NO_3_^−^–N [60].

### 2.4. Predicted Functional Genes in the Nitrogen Cycling in Filtration Systems

**Figure 5:**
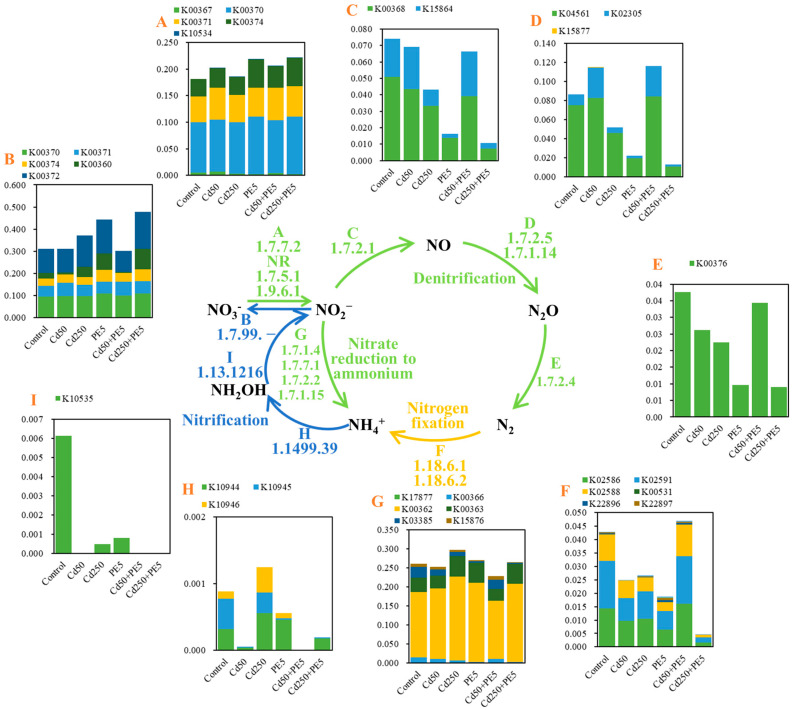
The reconstructed nitrogen-cycling processes in filtration systems under different contamination scenarios. (**A**–**I**) showed the relative abundance of genes in each pathway according to the KEEG database (Control represented control groups; Cd50 and Cd250 represented the concentration of Cd being 50 μg/L and 250 μg/L, respectively; PE5 represented the content of PE being 5%; + represented the combined contamination of Cd and PE).

Nitrogen-cycling processes in filtration systems under the single and combined contamination of PE and Cd were systematically characterized through KEGG pathway annotation (Figure 5). Functional genes associated with four key nitrogen transformation pathways were identified: nitrate reduction to ammonium, denitrification, nitrogen fixation, and nitrification. Genes-encoding enzymes (e.g., *narB* and *narG*) related to dissimilatory nitrate reduction to ammonium (DNRA) exhibited significantly higher relative abundance (0.351 ± 0.021) compared to those encoding enzymes (e.g., *narH*) related to assimilatory nitrate reduction to ammonium (ANRA) (0.103 ± 0.006) across all contamination scenarios. Distinct response patterns were observed among nitrogen-cycling genes under different contamination scenarios. Compared with genes encoding nitrite oxidase and nitrate reductase, the impact of PE and Cd contamination on other enzymes in the nitrification and denitrification was more intensive. The relative abundance of genes involved in the denitrification process under the combined contamination of PE5 and Cd50 was higher than those under PE5 single contamination and the combined contamination of PE5 and Cd250. This genetic response pattern aligns with the observed nitrogen removal efficiencies in Section 2.2, where the mean removal efficiency of NO_3_^−^–N was 16.37–43.16% higher than the other two contamination scenarios. Dose-dependent effects were particularly evident in Cd contamination. The genes involved in the denitrification process under Cd50 single contamination were more abundant than those under Cd250 single contamination, whereas those in the nitrification genes (e.g., *pmoA-amoA*, *pmoB-amoB* and *pmoC-amoC*) showed an opposite trend. This differential regulation may explain the contamination-specific removal efficiencies: the enhanced nitrification genes expression under Cd250 single contamination likely contributed to 17.74% higher NH_4_^+^–N removal compared to Cd50 single contamination in the sixth rainstorm event.

### 2.5. Synergistic and Antagonistic Effect Between PE and Cd

**Figure 6:**
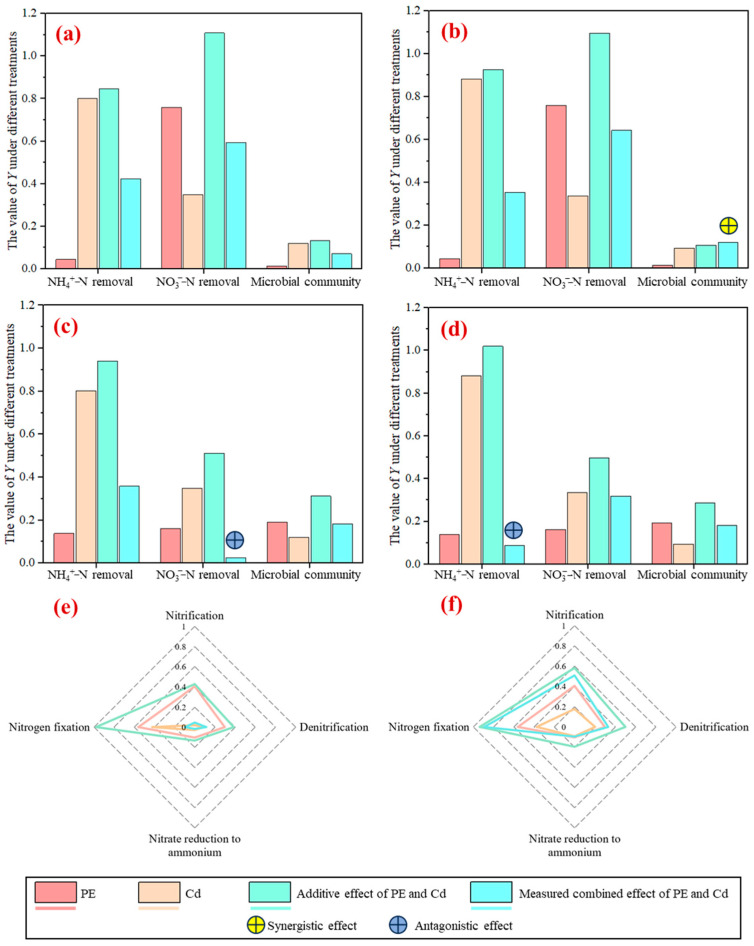
The value of *Y* in filtration systems under different contamination scenarios: PE content and Cd concentration were 0.1% and 50 μg/L, respectively (**a**); PE content and Cd concentration were 0.1% and 250 μg/L, respectively (**b**); PE content and Cd concentration were 5% and 50 μg/L, respectively (**c**,**e**); PE content and Cd concentration were 5% and 250 μg/L, respectively (**d**,**f**).

The combined effect of PE0.1 and Cd (both Cd50 and Cd250) on the removal of NO_3_^−^–N and NH_4_^+^–N were all between a synergistic and antagonistic effect, whereas PE5 showed an antagonistic effect with Cd under certain contamination levels (Figure 6). The combined effect of PE5 and Cd50 on the removal of NO_3_^−^–N was antagonistic, and PE5 also showed an antagonistic effect with Cd 250 on the removal of NH_4_^+^–N. The “dilution effects” might be one reason for the antagonistic effect between PE and Cd, which means that PE might increase Cd exchangeability and consequently attenuate the soil Cd retention [61]. PE can also be adsorbed by the filtration media, occupying the adsorption sites and facilitating the removal of Cd in the filtration system. Microplastics can also reduce Cd bioavailability by raising organic matter and humification levels in the filtration system [28,62]. PE0.1 and Cd250 posed synergistic effects on the microbial enrichment and diversity, and no synergistic effect or antagonistic effect was posed for other contamination scenarios. Microbes can colonize the surface of microplastics and the inhabited plastic ecosystems as the “plastisphere” [63]. The formation of the plastisphere might accelerate the adsorption of heavy metals, and the combined effect of microplastics and heavy metals could be strengthened [64]. No synergistic or antagonistic interactions were observed in the combined effects of PE and Cd across the four nitrogen metabolism pathways examined. However, the combined contamination of Cd250 and PE5 demonstrated higher impacts on three key metabolic pathways (denitrification, nitrogen fixation, and nitrification) compared to their individual effects. Notably, for nitrogen fixation processes, the measured combined effect of PE5 and Cd250 (*Y* value: 0.894) approximated that of their additive effect (*Y* value: 0.937). This observation suggests that PE and Cd may exert a synergistic effect on nitrogen fixation under specific environmental conditions. Zhang et al. [65] also found that the combined contamination of polyvinyl chloride and Cd altered the overall nitrogen-cycling capacity of the soil bacteria. Wang et al. [31] also highlighted synergistic effects between microplastics and Cd on influencing microbial functions.

To mitigate the impacts of Cd and microplastics, primary efforts should focus on implementing source reduction measures to control total emissions of Cd and microplastics generated by human activities, such as industrial and traffic sources. Effective interception of Cd and microplastics in stormwater runoff can prevent their entry into filtration systems, achievable through measures like sedimentation tanks or installing mesh filters. Additionally, real-time or periodic monitoring of Cd and microplastics in stormwater runoff, combined with establishing a risk early-warning mechanism, could further minimize the adverse effects of contaminated runoff on stormwater filtration systems.

## 3. Materials and Methods

### 3.1. Stormwater Filtration System Setup and Contamination Scenarios

**Figure 7:**
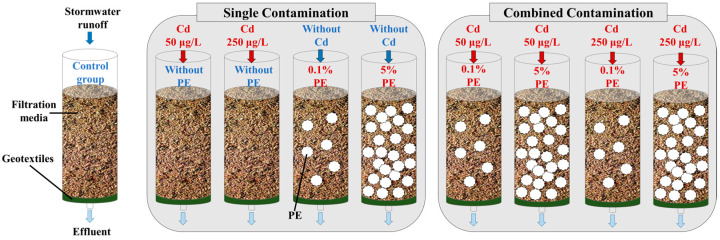
Structures of stormwater filtration systems and PE contamination scenario setting (Three stormwater filtration systems were used as three duplicates for each contamination scenario. A total of twenty-seven stormwater filtration systems were used. Red arrows and blue denoted Cd-containing and Cd-free influent, respectively).

Unaged PE was purchased from Dongguan Zhongxin Plastic Co., Ltd. (Dongguan, China). Unaged PE was passed through stainless steel sieves with mesh sizes of 230 μm and 250 μm, respectively. Particles within the range of 230–250 μm were collected and underwent an ultraviolet aging procedure, which was similar with that in our previous study [11]. Soil was collected in the campus of Tsinghua University; debris such as roots and stones were removed. The soil type is classified as cinnamon soil with a pH of approximately 6.5. Quartz sand was purchased from Xinshui Filter Material Co., Ltd. (Dongguan, China). Glass syringe barrels (200 mL capacity) were prepared by removing the plunger and needle. Stormwater filtration systems were constructed with glass syringes, soil, and quartz sand. A geotextile lining was installed in the syringe barrel under filtration media consisting of quartz sand and soil (Figure 7).

To establish microplastic contamination scenarios, aged polyethylene microplastics (PE, 100–300 μm) were homogenously mixed with filtration media at 0.1% and 5% (*w*/*w*) (PE0.1 and PE5, respectively). Each mixture (200 mL) was loaded into geotextile-lined syringes to construct filtration systems under varying PE contamination levels. To analyze the impact of Cd in stormwater runoff, Cd exposure scenarios were created using influent concentrations of 50 μg/L Cd^2+^ (Cd50) and 250 μg/L Cd^2+^ (Cd250); Cd leaching from soil is negligible [66,67]. Four single-contamination conditions were implemented: PE0.1, PE5, Cd50, and Cd250. Combined contamination was designated by “+” notation (e.g., Cd50 + PE0.1). Control systems contained neither PE nor Cd. Triplicate setups per condition yielded 27 filtration systems total.

To simulate the occurrence of organic matter, nitrogen, and phosphorus in real stormwater runoff, glucose, NH_4_Cl, KNO_3_, and KH_2_PO_4_ were added to synthetic stormwater for all rainfall events at concentrations of 300 mg/L, 0.8 mg/L, 1.2 mg/L, and 0.8 mg/L, respectively [68]. Six rainfall events were conducted at 5-day intervals over 30 days. The required water volume for per filtration system in each event was calculated as 509 mL using the following equation [69]:

(1) V=i×q×a×k×t

where *i* means comprehensive runoff coefficient, 0.9 in this study [70]; *q* means rainstorm intensity, 25 mm/min in this study [71]; *a* means stormwater filtration system area, 12.56 cm^2^ in this study; *k* means catchment-to-infiltrator area ratio, 1:15 in this study; *t* means rainstorm duration, 120 min in this study.

### 3.2. Nitrogen Removal Efficiency in Filtration Systems

Effluent samples were collected following each rainfall simulation, filtered through 0.45 μm nylon microfiltration membranes, and stored at 4 °C. NH_4_^+^–N and NO_3_^−^–N concentrations in influent and effluent were analyzed within 24 h using Nessler’s reagent spectrophotometry (HJ 535–2009) and ultraviolet spectrophotometry (HJ/T 346–2007), respectively.

The removal efficiency (*R*) of NH_4_^+^–N and NO_3_^−^–N was evaluated to assess the impact of contamination of PE and Cd in stormwater filtration systems as follows:

(2) $$R=\left(C_{in}-C_{e}\right)/C_{in}\times 100\%$$

where *C_in_* and *C_e_* mean the concentration of nitrogen species in the influent and effluent, respectively, both in mg/L in this study.

### 3.3. Microbial Community Detection

To investigate the single and combined effects of PE and Cd contamination on microbial community dynamics and elucidate their transformation mechanisms for nitrogen pollutants, filtration media samples were collected and subjected to microbial community analysis. For each filtration system, 5 g filtration media at the same depth (5 cm underneath the surface) on day 10, day 20, and day 30 were collected. Three duplicate samples of filtration media before the experiment were also collected. All filtration media samples were stored at −80 °C and analyzed within 10 days. The cryopreserved samples were transferred to Beijing BioGuoke Technology Co., Ltd. (Beijing, China) for comprehensive bacterial community characterization. Genomic DNA was extracted from the samples followed by PCR amplification targeting the V3–V4 hypervariable regions of the 16S rRNA gene. High-throughput sequencing was performed using the Illumina MiSeq platform (Illumina Inc., San Diego, CA, USA). Subsequent bioinformatics processing included clustering of operational taxonomic units (OTUs) at a 97% similarity threshold. This analytical approach enabled systematic evaluation of microbial community structure, including comparative analysis of taxonomic composition, species richness (Chao1 index), and diversity (Shannon index and Simpson index) across different contamination scenarios (Equations (3)–(5)) [72]. The relationship between conventional pollutants removal efficiencies and microbial communities was evaluated based on a redundancy analysis.

(3) $$Chao1=S_{obc}+\frac{F_{1}^{2}}{2F_{2}}$$

(4) $$Shannon=\exp(-p_{j}\ln(p_{j}))$$

(5) $$Simpson=\frac{1}{\sum_{j=1}^{J}p_{j}}$$

where *S_obc_* represents the number of species recorded in the sampled dataset; *F*_1_ and *F*_2_ mean the number of singleton species (species represented by exactly one individual in the sample) and doubleton species (species represented by exactly two individuals in the sample), respectively; *p_j_* means the relative abundance of species *j*; *J* is the count of individuals of species *j*.

### 3.4. Metagenomic Analysis

Six samples were collected from stormwater filtration systems to investigate nitrogen cycle responses under different contamination scenarios: control (uncontaminated), Cd50, Cd250, P5, Cd50 + PE5, and Cd250 + PE5. Each sample underwent standardized pretreatment procedures by Beijing BioGuoke Technology Co., Ltd. (Beijing, China). Sequencing-data quality control yielded clean reads of 80,739,310 (Control), 85,743,304 (Cd50), 74,090,590 (Cd250), 76,519,960 (P5), 86,908,382 (Cd50 + PE5), and 84,186,622 (Cd250 + PE5), respectively. Processed sequences were functionally annotated using the KEGG Orthology database. Nitrogen-cycle-related functional genes were specifically extracted using the KEGG nitrogen metabolism pathway map (ko00910) as a reference framework.

### 3.5. Analysis on the Interaction Between PE and Cd

Interactions between PE and Cd on NH_4_^+^–N, NO_3_^−^–N, microbial diversity and richness, and functional genes abundance in key metabolic pathways were estimated as follows:

(6) $$X_{i,additive}=\left|X_{i,Cd}-X_{i,control}\right|+\left|X_{i,PE}-X_{i,control}\right|$$

where *X_i_* represents the different parameters (e.g., NH_4_^+^–N removal, NO_3_^−^–N removal, Chao1 index, Shannon index, Simpson index, and functional genes abundance); *X_i,additive_* means the change in parameter *X_i_* under the additive effect of Cd and PE treatment; *X_i,control_*, *X_i,Cd_*, and *X_i,PE_* represent the value of *X_i_* under no Cd/PE treatment (control group), Cd treatment, or PE treatment.

In this study, the percentage change in *X* under Cd contamination (*Y_i,Cd_*), PE contamination (*Y_i,PE_*), and combined contamination of Cd and PE (*Y_i,additive_*) was used to better compare the effect among these contamination scenarios; the formula is as follows:

(7) $$Y_{i,additive}=Y_{i,Cd}+Y_{i,PE}=\frac{\left|X_{i,Cd}-X_{i,control}\right|}{X_{i,control}}+\frac{\left|X_{i,PE}-X_{i,control}\right|}{X_{i,control}}$$

The combination effect of PE and Cd (*Y_i,PE+Cd_*) treatment higher than *Y_i,additive_* indicates a synergistic effect between them, and *Y_i,PE+Cd_* lower than both *Y_i,PE_* and *Y_i,Cd_* indicates an antagonistic effect between them.

## 4. Conclusions

PE exhibited inhibitory effects on Cd takeaway efficiency in filtration systems, with the magnitude of inhibition showing a positive correlation with PE concentration. Both individual and combined contamination scenarios of PE and Cd demonstrated contrasting impacts on nitrogen removal—enhancing NH_4_^+^–N removal while suppressing NO_3_^−^–N elimination. The beneficial effect on NH_4_^+^–N removal was less pronounced under PE contamination compared to Cd exposure, and PE demonstrated stronger inhibition on NO_3_^−^–N removal than Cd. Notably, high-level PE contamination (PE5) in Cd-containing systems enhanced NO_3_^−^–N removal efficiency, contrasting with the inhibitory effect observed under low-level PE contamination (PE0.1). During the sixth rainstorm event, the mean NO_3_^−^–N removal efficiency under combined Cd50 and PE5 contamination exceeded that of other contamination scenarios by 1.04 to 34.68 folds.

The microbial community analysis revealed temporal dynamics, with the combined contamination of PE and Cd initially reducing microbial richness and diversity (Day 10) followed by recovery (Day 30). Distinct microbial selection patterns emerged between contaminants: Cd single contamination increased Proteobacteria abundance (>70.14%) relative to controls (62.97%), while PE exposure substantially reduced Proteobacteria representation (<43.12%). Six key genera associated with nitrogen removal were identified: *Unclassified_o_Saccharimonadales*, *Unidentified_o_Saccharimonadales*, *Enterobacter*, *Unclassified_f_Enterobacteriaceae*, *Aeromonas*, and *Cloacibacterium*.

Functional gene analysis identified four critical nitrogen transformation pathways: nitrate reduction to ammonium, denitrification, nitrogen fixation, and nitrification. Enzymes associated with DNRA (e.g., narB, *narG*) demonstrated significantly higher relative abundance (0.351 ± 0.021) compared to ANRA-associated enzymes (e.g., narH; 0.103 ± 0.006). Contamination effects were most pronounced on nitrification/denitrification enzymes beyond nitrite oxidase and nitrate reductase. Concentration-dependent responses were particularly evident for Cd exposure, with denitrification genes under Cd50 exceeding those in Cd250 systems. Enhanced denitrification gene abundance under combined contamination of Cd50 and PE5 relative to PE5 single contamination and combined contamination of Cd250 and PE5 correlated with observed NO_3_^−^–N removal efficiency differences.

## Figures and Tables

**Table 1:** Values of richness and diversity indexes.

Scenario	Chao1			Shannon			Simpson		
	10th Day	20th Day	30th Day	10th Day	20th Day	30th Day	10th Day	20th Day	30th Day
Control	754.06	673.55	784.70	7.08	5.94	7.06	0.98	0.90	0.98
Cd50	493.08	487.36	661.23	6.10	5.61	6.55	0.94	0.93	0.96
Cd250	401.37	501.00	778.57	5.71	6.30	7.38	0.91	0.95	0.98
PE0.1	514.78	648.00	1006.28	5.75	6.79	7.75	0.88	0.97	0.98
Cd50 + PE0.1	492.90	576.73	894.72	6.89	6.97	7.78	0.97	0.98	0.98
Cd250 + PE0.1	374.59	810.94	463.53	5.39	7.18	5.81	0.87	0.98	0.94
PE5	375.62	524.03	489.27	5.76	6.21	4.87	0.95	0.96	0.83
Cd50 + PE5	497.09	491.50	376.26	6.27	6.25	4.66	0.96	0.96	0.88
Cd250 + PE5	386.50	608.69	344.67	5.83	6.51	5.00	0.94	0.97	0.91

## Data Availability

Data available on request due to restrictions.

## Supplementary Materials

The following supporting information can be downloaded at https://www.mdpi.com/article/10.3390/molecules30071464/s1, Table S1: Concentrations of inorganic elements in aged PE (in μg/g).

## Abbreviations

The following abbreviations are used in this manuscript:

PE	Polyethylene microplastic
Cd	Cadmium
NH_4_^+^–N	Ammonium nitrogen
NO_3_^−^–N	Nitrate nitrogen
DNRA	Dissimilatory nitrate reduction to ammonium
ANRA	Assimilatory nitrate reduction to ammonium

## References

[1] Shahzad H., Myers B., Boland J., Hewa G., Johnson T. (2022). Stormwater runoff reduction benefits of distributed curbside infiltration devices in an urban catchment. Water Res..

[2] Zhang K., Parolari A.J. (2022). Impact of stormwater infiltration on rainfall-derived inflow and infiltration: A physically based surface–subsurface urban hydrologic model. J. Hydrol..

[3] Zhao J., Shu L., Wu M., Han J., Luo S., Tang J. (2023). Stormwater runoff pollution control performance of permeable concrete pavement and constructed wetland combined system: Toward on-site reuse. Water Sci. Technol..

[4] Shokri M., Kibler K.M., Hagglund C., Corrado A., Wang D., Beazley M., Wanielista M. (2021). Hydraulic and nutrient removal performance of vegetated filter strips with engineered infiltration media for treatment of roadway runoff. J. Environ. Manag..

[5] Fan G., Ning R., Huang K., Wang S., You Y., Du B., Yan Z. (2021). Hydrologic characteristics and nitrogen removal performance by different formulated soil medium of bioretention system. J. Clean. Prod..

[6] Smyth K., Drake J., Li Y., Rochman C., Van Seters T., Passeport E. (2021). Bioretention cells remove microplastics from urban stormwater. Water Res..

[7] Lin Y., Wang Y., Ho Y.W., Fang J.K.H., Li Y. (2024). Characterization and ecological risks of microplastics in urban road runoff. Sci. Total Environ..

[8] Lee Y.K., Yoo H.Y., Ko K.S., He W., Karanfil T., Hur J. (2022). Tracing microplastic (MP)-derived dissolved organic matter in the infiltration of MP-contaminated sand system and its disinfection byproducts formation. Water Res..

[9] Kang W., Sun S., Hu X. (2023). Microplastics trigger the Matthew effect on nitrogen assimilation in marine diatoms at an environmentally relevant concentration. Water Res..

[10] Liu Z., Cai L., Dong Q., Zhao X., Han J. (2022). Effects of microplastics on water infiltration in agricultural soil on the Loess Plateau, China. Agric. Water Manag..

[11] Men C., Ma Y., Liu J., Zhang Y., Li Z., Zuo J. (2024). The difference between tire wear particles and polyethylene microplastics in stormwater filtration systems: Perspectives from aging process, conventional pollutants removal and microbial communities. Environ. Pollut..

[12] Jiang X., Chen H., Liao Y., Ye Z., Li M., Klobučar G. (2019). Ecotoxicity and genotoxicity of polystyrene microplastics on higher plant Vicia faba. Environ. Pollut..

[13] Feng H., Xing X., Du J., Jiao S., Yu M., Wang W. (2025). Concentration- and size-dependent influences of microplastics on soil hydraulic properties and water flow. Eur. J. Soil Sci..

[14] Li K., Xiu X., Hao W. (2024). Microplastics in soils: Production, behavior process, impact on soil organisms, and related toxicity mechanisms. Chemosphere.

[15] Yang J., Zhang T., Ma S., Shang J., Li L., Ning Y., Zhao X. (2025). Enhancing microplastic removal and nitrogen mitigation in constructed wetlands: An earthworm-centric perspective. J. Hazard. Mater..

[16] Zhou Z., Hua J., Xue J., Yu C. (2024). Differential impacts of polyethylene microplastic and additives on soil nitrogen cycling: A deeper dive into microbial interactions and transformation mechanisms. Sci. Total Environ..

[17] Liu Y., Chen S., Zhou P., Li H., Wan Q., Lu Y., Li B. (2024). Differential impacts of microplastics on carbon and nitrogen cycling in plant-soil systems: A meta-analysis. Sci. Total Environ..

[18] Zhao Y., Hu Z., Hao Z., Xie H., Liu D., Yan P., Xu H., Wu H., Zhang J. (2024). Revealing the size effect mechanisms of micro(nano)plastics on nitrogen removal performance of constructed wetland. J. Hazard. Mater..

[19] Göbel P., Dierkes C., Coldewey W.G. (2007). Storm water runoff concentration matrix for urban areas. J. Contam. Hydrol..

[20] Yu J., Yu H., Fang H., Lei M., Li S., Chi J. (2014). Pollution characteristics of lead, zinc, arsenic, and cadmium in short-term storm water roof runoff in a suburban area. Toxicol. Environ. Chem..

[21] Soltaninia S., Eskandaripour M., Ahmadi Z., Ahmadi S., Eslamian S. (2024). The hidden threat of heavy metal leaching in urban runoff: Investigating the long-term consequences of land use changes on human health risk exposure. Environ. Res..

[22] Muthanna T.M., Viklander M., Blecken G., Thorolfsson S.T. (2007). Snowmelt pollutant removal in bioretention areas. Water Res..

[23] Elrys A.S., Wen Y., Feng D., El-Mekkawy R.M., Kong M., Qin X., Lu Q., Dan X., Zhu Q., Tang S. (2025). Cadmium inhibits carbon and nitrogen cycling through soil microbial biomass and reduces soil nitrogen availability. J. Hazard. Mater..

[24] Wang G., Yu G., Chi T., Li Y., Zhang Y., Wang J., Li P., Liu J., Yu Z., Wang Q. (2023). Insights into the enhanced effect of biochar on cadmium removal in vertical flow constructed wetlands. J. Hazard. Mater..

[25] Wu S., Cai C., Wang W., Bao M., Huang J., Dai Y., Wang Y., Cheng S. (2024). The interaction of microplastic and heavy metal in bioretention cell: Contributions of water-soil-plant system. Environ. Pollut..

[26] Jakubowicz P., Fitobor K., Gajewska M., Drewnowska M. (2022). Detection and removal of priority substances and emerging pollutants from stormwater: Case study of the Kolobrzeska collector, Gdansk, Poland. Sustainability.

[27] Jiang C., Li J., Hu Y., Yao Y., Li H. (2022). Construction of water-soil-plant system for rainfall vertical connection in the concept of sponge city: A review. J. Hydrol..

[28] Xu Z., Bai X., Li Y., Weng Y., Li F. (2023). New insights into the decrease in Cd^2+^ bioavailability in sediments by microplastics: Role of geochemical properties. J. Hazard. Mater..

[29] Huang F., Chen L., Yang X., Jeyakumar P., Wang Z., Sun S., Qiu T., Zeng Y., Chen J., Huang M. (2024). Unveiling the impacts of microplastics on cadmium transfer in the soil-plant-human system: A review. J. Hazard. Mater..

[30] Guo X., Wang J. (2021). Projecting the sorption capacity of heavy metal ions onto microplastics in global aquatic environments using artificial neural networks. J. Hazard. Mater..

[31] Wang Y., Zhang F., Yang L., Zhang G., Wang H., Zhu S., Zhang H., Guo T. (2025). Synergy of plastics and heavy metals weakened soil bacterial diversity by regulating microbial functions in the Qinghai-Tibet Plateau. J. Hazard. Mater..

[32] Lan T., Dong X., Liu S., Zhou M., Li Y., Gao X. (2024). Coexistence of microplastics and Cd alters soil N transformation by affecting enzyme activity and ammonia oxidizer abundance. Environ. Pollut..

[33] Wang Q.Y., Wang Q.R., Wang T.Y., Zhang S.Q., Yu H.W. (2024). Impacts of polypropylene microplastics on the distribution of cadmium, enzyme activities, and bacterial community in black soil at the aggregate level. Sci. Total Environ..

[34] Zeb A., Liu W., Meng L., Lian J., Wang Q., Lian Y., Chen C., Wu J. (2022). Effects of polyester microfibers (PMFs) and cadmium on lettuce (*Lactuca sativa*) and the rhizospheric microbial communities: A study involving physio-biochemical properties and metabolomic profiles. J. Hazard. Mater..

[35] Zhang S., Han B., Sun Y., Wang F. (2020). Microplastics influence the adsorption and desorption characteristics of Cd in an agricultural soil. J. Hazard. Mater..

[36] Meng Z., Wu J., Huang S., Xin L., Zhao Q. (2024). Competitive adsorption behaviors and mechanisms of Cd, Ni, and Cu by biochar when coexisting with microplastics under single, binary, and ternary systems. Sci. Total Environ..

[37] Qiu Y., Zhou S., Zhang C., Zhou Y., Qin W. (2022). Soil microplastic characteristics and the effects on soil properties and biota: A systematic review and meta-analysis. Environ. Pollut..

[38] Zhou Z., Hua J., Xue J. (2023). Polyethylene microplastic and soil nitrogen dynamics: Unraveling the links between functional genes, microbial communities, and transformation processes. J. Hazard. Mater..

[39] Porter S.K., Scheckel K.G., Impellitteri C.A., Ryan J.A. (2004). Toxic metals in the environment: Thermodynamic considerations for possible immobilization strategies for Pb, Cd, As, and Hg. Crit. Rev. Environ. Sci. Technol..

[40] Ding W., Qin H., Wang F., Xia C. (2024). Leaching sources and mechanisms of different nitrogen species from bioretention systems. Water Res..

[41] Yu X., Zhao J., Liu X., Sun L., Tian J., Wu N. (2021). Cadmium pollution impact on the bacterial community structure of arable soil and the isolation of the cadmium resistant bacteria. Front. Microbiol..

[42] Liu H., Yang Y., Yang Y., Zhong X., Lv J. (2022). Dynamics of fungal and bacterial communities in different types of soil ageing with different dosages of cadmium. Ecotoxicol. Environ. Saf..

[43] Sen S.K., Raut S., Dora T.K., Mohapatra P.K. (2014). Contribution of hot spring bacterial consortium in cadmium and lead bioremediation through quadratic programming model. J. Hazard. Mater..

[44] Matyar F., Kaya A., Dinçer S. (2008). Antibacterial agents and heavy metal resistance in Gram-negative bacteria isolated from seawater, shrimp and sediment in Iskenderun Bay, Turkey. Sci. Total Environ..

[45] Qiu G., Wang Q., Wang Q., Wang T., Kang Z., Zeng Y., Yang X., Song N., Zhang S., Han X. (2023). Effects of polyethylene microplastics on properties, enzyme activities, and the succession of microbial community in Mollisol: At the aggregate level. Environ. Res..

[46] Bajo K., Romano R., Kolvenbach B., Nazemi S.A., Shahgaldian P., Corvini P.F.X., Fava F., Raddadi N. (2024). Biodegradation of untreated plasticizers-free linear low-density polyethylene films by marine bacteria. Mar. Pollut. Bull..

[47] Szumigaj J., Zakowska Z., Klimek L. (2008). Exopolysaccharide production by Bacillus strains colonizing packaging foils. Pol. J. Microbiol..

[48] Gupta K.K., Sharma K.K., Chandra H. (2022). Micrococcus luteus strain CGK112 isolated from cow dung demonstrated efficient biofilm-forming ability and degradation potential toward high-density polyethylene (HDPE). Arch. Microbiol..

[49] Hong J., Ko D., Hwang Y. (2020). Disulfide polymer grafted polypropylene/polyethylene filter media for selective cadmium removal. J. Hazard. Mater..

[50] Guo J.J., Li F., Xiao H.C., Liu B.L., Feng L.N., Yu P.F., Meng C., Zhao H.M., Feng N.X., Li Y.W. (2023). Polyethylene and polypropylene microplastics reduce chemisorption of cadmium in paddy soil and increase its bioaccessibility and bioavailability. J. Hazard. Mater..

[51] Yu H., Zhang Z., Zhang Y., Fan P., Xi B., Tan W. (2021). Metal type and aggregate microenvironment govern the response sequence of speciation transformation of different heavy metals to microplastics in soil. Sci. Total Environ..

[52] Deng R., Huang D., Xue W., Lei L., Zhou C., Chen S., Wen X., Liu X. (2020). How does the microenvironment change during the stabilization of cadmium in exogenous remediation sediment?. J. Hazard. Mater..

[53] Tudi M., Yang L., Yu J., Wei B., Xue Y., Wang F., Li L., Yu Q.J., Ruan H.D., Li Q. (2023). Leaching characteristics and potential risk of heavy metals from drip irrigation pipes and mulch substrate in agricultural ecosystems. Sci. Total Environ..

[54] Xu W., Lam C., Wang Y., Wan S.H., Ho P.H., Myung J., Yung C.C.M. (2025). Temporal succession of marine microbes drives plastisphere community convergence in subtropical coastal waters. Environ. Pollut..

[55] Veach A.M., Zeglin L.H. (2020). Historical drought affects microbial population dynamics and activity during soil drying and re-wet. Microb. Ecol..

[56] Zhu H., Li W., Chen X., Mu H., Hu K., Ren S., Peng Y., Zhao R., Wang Y. (2023). Effects of sponge iron dosage on nitrogen removal performance and microbial community structure in sequencing batch reactors. Bioresour. Technol..

[57] Xue Z., Zhang T., Sun Y., Yin T., Cao J., Fang F., Feng Q., Luo J. (2022). Integrated moving bed biofilm reactor with partial denitrification-anammox for promoted nitrogen removal: Layered biofilm structure formation and symbiotic functional microbes. Sci. Total Environ..

[58] Miyakoshi M., Morita T., Kobayashi A., Berger A., Takahashi H., Gotoh Y., Hayashi T., Tanaka K. (2022). Glutamine synthetase mRNA releases sRNA from its 3′UTR to regulate carbon/nitrogen metabolic balance in Enterobacteriaceae. eLife.

[59] Dong Y., Zhang X., Zou L.A., Wang Z., Chen F., Li L. (2022). Isolation and identification of a cold-tolerant and aerobic denitrifying bacterium *Aeromonas* sp. and optimization of denitrification conditions. Acta Microbiol. Sin..

[60] Ren X., Niu H., Yuan J., Duan Y., Fan X. (2024). Construction of acid-resistant denitrifying mixed bacterial consortium and enhanced biological nitrogen removal. China Environ. Sci..

[61] Wang F., Zhang X., Zhang S., Zhang S., Adams C.A., Sun Y. (2020). Effects of co-contamination of microplastics and Cd on plant growth and Cd accumulation. Toxics.

[62] Machado A.A.d.S., Lau C.W., Kloas W., Bergmann J., Bachelér J.B., Faltin E., Becker R., Goerlich A.S., Rillig M.C. (2019). Microplastics can change soil properties and affect plant performance. Environ. Sci. Technol..

[63] Zhu D., Ma J., Li G., Rillig M.C., Zhu Y.G. (2022). Soil plastispheres as hotspots of antibiotic resistance genes and potential pathogens. ISME J..

[64] Wang C., Wang L., Ok Y.S., Tsang D.C.W., Hou D. (2022). Soil plastisphere: Exploration methods, influencing factors, and ecological insights. J. Hazard. Mater..

[65] Zhang Y., Zhao S.Y., Zhang R.H., Li B.L., Li Y.Y., Han H., Duan P.F., Chen Z.J. (2024). Screening of plant growth-promoting rhizobacteria helps alleviate the joint toxicity of PVC+Cd pollution in sorghum plants. Environ. Pollut..

[66] Lu X., Guo Y. (2018). Detection of trace cadmium in soil by potassium iodide-Cd(II)-Rhodamine B ion association spectrophotometry. Appl. Chem. Ind..

[67] Shafiq M., Iqbal M.Z., Arayne M.S., Athar M. (2012). Biomonitoring of heavy metal contamination in *Pongamia pinnata* and *Peltophorum pterocarpugrowing* in the polluted environment of Karachi, Pakistan. J. Appl. Bot. Food Qual..

[68] You Z., Zhang L., Pan S.Y., Chiang P.C., Pei S., Zhang S. (2019). Performance evaluation of modified bioretention systems with alkaline solid wastes for enhanced nutrient removal from stormwater runoff. Water Res..

[69] Zhang H., Zhang X., Liu J., Zhang L., Li G., Zhang Z., Gong Y., Li H., Li J. (2022). Coal gangue modified bioretention system for runoff pollutants removal and the biological characteristics. J. Environ. Manag..

[70] He W., Lin X., Shi Z., Yu J., Ke S., Lu X., Deng Z., Wu Y., Wang L., He Q. (2022). Nutrient removal performance and microbial community analysis of amended bioretention column for rainwater runoff treatment. J. Clean. Prod..

[71] Wang Y., Hu S., Tu J., Zeng Q., Zhang H., Huang X., Gong Y., Zhang H. (2021). Verification and evaluation of quantitative precipitation forecast for Guangdong Province during 2016–2020. J. Trop. Meteorol..

[72] Huang C., Zhang W., Shen Z., Li M., Yin J., Tang Y., Zhou X., Zhu X., Sun Z. (2024). The association between alpha diversity of gut microbiota, neuroimaging markers and cognitive function in cerebral small vessel disease. Brain Res..

