# Supplementary material for: Single and Combined Effects of Aged Polyethylene Microplastics and Cadmium on Nitrogen Species in Stormwater Filtration Systems: Perspectives from Treatment Efficiency, Key Microbial Communities, and Nitrogen Cycling Functional Genes
Source: Molecules. 2025 Mar 26;30(7):1464. doi: 10.3390/molecules30071464 (PMC11990492; doi:10.3390/molecules30071464)
Supplement: Supplementary file 1 [file molecules-30-01464-s001.zip › molecules-3517745-supplementary.pdf]

# Single and combined effects of aged polyethylene microplastics and cadmium on nitrogen species in stormwater filtration systems: Perspectives from treatment efficiency, key microbial communities, and nitrogen cycling functional genes

## Supplementary Materials

**Table S1 Concentrations of inorganic elements in aged PE (in μg/g)**

| Particles | Ru | Pd | Sn | Sb | Te | Hf | Ir | Pt | Au | Na | Mg | P |
|---|---|---|---|---|---|---|---|---|---|---|---|---|
| PE | 0.170 | 1.198 | 59.368 | 0.160 | 126.066 | 98.491 | 0.425 | 0.915 | 1.302 | 374.925 | 282.028 | n.d. |
| Particles | K | Ca | Li | Be | Al | V | Cr | Mn | Fe | Co | Ni | Cu |
| PE | 33.547 | 360.792 | 9.840 | n.d. | 121.528 | 0.160 | 2.340 | 5.717 | 57.085 | 0.057 | 1.802 | 0.245 |
| Particles | Zn | Ga | As | Se | Ag | Cd | Cs | Ba | Tl | Pb | Y | La |
| PE | 77.632 | n.d. | 0.566 | 7.472 | 0.028 | n.d. | n.d. | 0.698 | 0.047 | 0.613 | 0.047 | 0.481 |
| Particles | Ce | Pr | Nd | Sm | Eu | Gd | Tb | Dy | Ho | Er | Tm | Yb |
| PE | 0.377 | 0.066 | 0.104 | 0.047 | 0.057 | n.d. | n.d. | 0.066 | 0.057 | 0.066 | 0.057 | 0.057 |
| Particles | Lu | B | Ti | Nb | Mo | W | Si | Sum | - | - | - | - |
| PE | n.d. | n.d. | n.d. | 0.123 | 0.377 | 0.557 | 44.340 | 1.628E+06 | - | - | - | - |

Note: n.d. means the concentration is below the detection limit.
